# Breastfeeding duration of different age groups and its associated factors among Chinese women: a cross-sectional study

**DOI:** 10.1186/s13006-019-0212-2

**Published:** 2019-05-09

**Authors:** Kun Tang, Yaqian Liu, Ke Meng, Li Jiang, Shihui Tan, Yuning Liu, Jiawen Chen

**Affiliations:** 1 Research Center for Public Health, Tsinghua University School of Medicine, Haidian District, Beijing, 10084 China; 2 School of Public Policy and Management, Tsinghua University, Haidian District, Beijing, 100084 China; 3 Peking University People’s Hospital, No. 11 Xizhimen South Ave., Xicheng District, Beijing, 100044 China; 4 School of Basic Medical Sciences, Peking University Health Science Center, 38 Xueyuan Road, Haidian District, Beijing, 100191 China; 5 Department of Global Health and Population, Harvard T.H. Chan School of Public Health, 677 Huntington Avenue, Boston, MA 02115 USA; 6 School of Nursing, Peking University Health Science Center, 38 Xueyuan Road, Haidian District, Beijing, 100191 China

**Keywords:** Age difference, Breastfeeding duration, Reproductive factors, Education

## Abstract

**Background:**

The current situation of breastfeeding in China has been discussed in many articles, and a declining trend of breastfeeding duration has been confirmed. The associations between various socioeconomic, reproductive factors and breastfeeding duration have been discussed as well. However, there remains a lack of evidence on breastfeeding duration amongst different age groups.

**Methods:**

Data was extracted from the baseline of a large cohort study: a sample of 300,000 adult women born in the 1930s’ through 1970s’ from 10 geographically distinct regions was obtained. The breastfeeding duration was assumed by breastfeeding duration of the first child. Different age groups were categorized by 10-year age groups, and they were born in 1930–1938, 1939–1948, 1949–1958, 1959–1968, and 1969–1974. Multivariable linear regression was used to evaluate the associations between breastfeeding duration and sociodemographic, and reproductive factors (i.e.: different age groups, education, household size, use of oral contraceptive pills, age at menarche, and age at first birth).

**Results:**

The mean breastfeeding duration (Standard Deviation) of women born in 1930–1938, 1939–1948, 1949–1958, 1959–1968, and 1969–1974 were 15.4 (9.2), 14.8 (8.2), 14.7 (8.9), 12.8 (7.2), 13.1 (7.2) months, respectively. Younger age groups, higher levels of education, and use of oral contraceptive pills were negatively associated with breastfeeding duration. In particular, the negative association with higher levels of education (for urban regions: β_middle school_ = − 1.3, β_high school & above_ = − 1.6; for rural regions: β_middle school_ = − 0.6, β_high school & above_ = − 1.2; all *p* < 0.0001) was found significant in both rural and urban areas, household size (β = − 0.1, *p* < 0.0001) and age at first birth (β = − 0.2, *p* < 0.0001) were negatively associated with breastfeeding duration only in urban areas, but were all positively associated with breastfeeding duration in rural areas.

**Conclusions:**

This research demonstrated that, among Chinese women, younger age groups and higher levels of education were negatively associated with breastfeeding duration. Considering the declining trend of breastfeeding duration in China, healthcare providers need to continue advocating for breastfeeding practices. We also call for collaboration with various sectors and concerned groups to actions that promote breastfeeding-friendly environment and measures.

## Background

Despite the well-established evidence on the importance of breastfeeding, previous studies have documented a downward trend of breastfeeding in China. Over the past 40 years, the rate of ‘ever breastfeeding’ declined in both urban and rural areas between the 1960s’ and 1970s’ [1], and the breastfeeding rate reached the lowest level in the 1980s’ [2]. It remained at a low level in Beijing until the 1990s’. The rate of ‘any breastfeeding’ at 4 months was nearly 60% while the rates of ‘full breastfeeding’ at 4 months were 62.8, 56.9, 61.3 and 55.9% in 1989, 1990, 1991 and 1992 respectively. In a retrospective study among 826 women in Tianjin, China, it was approximated that the breastfeeding duration was 25 months before 1932, 24 months from 1933 to 1942, 20 months from 1943 to 1952, 17 months from 1953 to 1972 and 13 months from 1973 to 1982 [3]. A literature review has suggested a consistent decline in breastfeeding throughout generations hence warrants careful study of this trend.

Reasons of the declining trend of breastfeeding practice mentioned above are multi-factorial in essence. Studies have revealed findings on social, economic, and cultural influences. While China has experienced unprecedented development in the past decades, the rapid changes in lifestyle will inevitably influence women’s breastfeeding behaviors. Yet, evidence on different age groups and breastfeeding is still largely missing [2]. A proper understanding of the relationship between demographic, social, economic, lifestyle factors and individual breastfeeding behavior will not only enable health interventions, but also provide important evidence for other countries who are at a similar development stage. By using the data from the baseline of a large cohort study, the present study aims to explore factors associated with different age groups in breastfeeding duration in urban and rural regions, in order to generate evidence enabling further understanding on the transition of breastfeeding.

## Methods

### Baseline survey

Data were extracted from the baseline survey of a large cohort study [4]. In brief, over 300,000 women from 1737 communities in 10 geographically defined areas in China aged between 30 and 79 were enrolled in the survey between 2004 and 2008. The 10 regions were selected to provide approximately equal coverage of rural (Gansu, Henan, Sichuan, Hunan, and Zhejiang) and urban (Harbin, Qingdao, Suzhou, Liuzhou, and Haikou) provinces. The selection of the 10 regions was based on local disease patterns and exposure to certain risk factors, population stability, quality of death and disease registries, local commitment and capacity. A Regional Coordinating Centre (RCC) and survey team, comprised of about 15 full-time staff with medical qualifications and fieldwork experience, were established in each of the 10 study areas. A quality control (QC) survey was completed in a selected community within a few weeks after the initial baseline survey. About 3% of the participants was selected randomly from that community with repeat questionnaire and measures on selected items. During the course of the survey, regular central monitoring was also undertaken to assess recruitment rate, the distribution of certain key variables, and consistency of the data collected, both overall and by individual staff. On-site monitoring visits were also undertaken every 6 months by provincial Centre for Disease Control and Prevention (CDC) staff and annually by Oxford/Beijing staff. The women involved in the research all had at least one child. Women who had children but never breastfed were also included. Community leaders or health workers approached potential participants in person. The electronic questionnaire was administered by trained staff, information on sociodemographic characteristics, socioeconomic status, lifestyle habits, reproductive and medical history were collected. Blood samples and physical measurements of each participant were collected using the standardized methods [5].

National and international ethical approval was obtained from the Ethical Review Committee of the Chinese Centre for Disease Control and the Institutional Review Boards at Oxford University. All study participants provided written informed consent.

### Assessment of breastfeeding duration

In the baseline survey questionnaire, participants were asked about breastfeeding history of each live birth. The breastfeeding duration of the first child was used as mothers’ breastfeeding duration.

### Exposure

In this study, women were born in 1930 through 1974. Due to difference in the social, economic, and political backgrounds in the years involved that may affect breastfeeding duration, the study population was categorized into five age groups by 10-year difference: 1930–1938, 1939–1948, 1949–1958, 1959–1968 and 1969–1974.

### Other covariates

Demographic characteristics including education (no formal school, primary school, middle school, high school & university & technical school) and household size (≤ 3, 3–5 and > 5 persons) were gathered from in-person interviews. Reproductive health information including number of live births (1, 2 and > 2), age at menarche (< 15 and ≥ 15 years old), use of oral contraceptive pills (never used and used) and age at first birth (≤ 21, 21–25 and > 25 years old) were also collected from in-person interviews. Each Body Mass Index (BMI) at age 25 (< 18.5, 18.5–24, > 24 kg/m^2^) was calculated using weight at age 25 and the present height.

### Statistical analysis

Descriptive analyses were conducted to present the basic characteristics of the study population. The continuous variables were presented as means Standard Deviations (SDs) and the categorical variables as percentages to describe the participants. Analyses of the association between breastfeeding duration and different age groups were conducted separately for urban and rural areas and categorized by 10-year age group. A general linear model was used to estimate the mean values and *p* - values of various socioeconomic factors in different regions and age groups of breastfeeding duration, adjusted for level of education, use of oral contraceptive pills, BMI at age 25 and age at menarche and at first birth. Linear least squares regression method was used to adjust geometric mean values and 95% confidence intervals (CI) of breastfeeding duration in different age groups by education, adjusted for level of education, use of oral contraceptive pills, age at menarche, age at first birth, and BMI at age 25. All analyses were performed using SAS version 9.4 (SAS Institute, Cary, North Carolina, USA).

## Results

The main demographic and reproductive history information of the 301,744 women participated in the study are given in Table 1. The mean ages (SD) of women born in the 1930s’, 1940s’, 1950s’, 1960s’, and 1970s’ were 70.5 (2.4), 61.6 (3.1), 52.0 (3.0), 41.8 (2.9), and 35.7 (1.4) respectively. The rural residency rates of women in these five age groups were 43.5, 53.3, 55.5, 58.8, and 60.1%. As the birth years increased, the rate of women with highest level of education at primary school fell from 85.8 to 38.5%, while the rate of women with middle school education grew from 6.8 to 36.7% and the rate of women with educational level higher than high school increased from 7.4 to 24.7%. Menarche tended to occur earlier as the age group increased, and the mean age at menarche fell from 16.0 (2.0) to 14.3 (1.6). Nearly 90% of all women had never used oral contraceptive pills in each group. The mean ages at first birth were 22.0 (3.6), 22.5 (3.4), 24.0 (3.2), 23.5 (2.7), 23.8 (2.9) for each group. The majority of women gave their first births between age 21 and 25, with an increasing rate from 30.4 to 50.8%. In addition, the number of live births dropped sharply. Nearly 90% of women born in the 1930s’ had more than two children, while more than 90% born in the 1970s’ had only one or two children.

**Table 1:** Basic characteristic of participants by birth cohort (*n* = 301,744)

	1930–1938 (*n* = 26,723)	1939–1948 (*n* = 57,571)	1949–1958 (*n* = 99,680)	1959–1968 (*n* = 92,452)	1969–1974 (*n* = 25,318)
Mean age/years, (SD)	70.5 (2.4)	61.6 (3.1)	52.0 (3.0)	41.8 (2.9)	35.7 (1.4)
Rural residency, %	43.6	53.3	55.5	58.8	60.1
Urban residency, %	56.5	46.7	44.5	41.2	40.0
Highest education, %					
Primary school	85.9	75.0	63.4	34.7	38.5
Middle school	6.8	14.8	21.7	38.2	36.7
High school and above	7.3	10.3	14.8	27.1	24.7
Household size, %					
≤3 people	59.0	50.6	44.8	46.2	44.7
3–5 people	30.3	34.4	40.0	45.8	46.2
>5 people	10.7	15.0	15.2	8.1	9.1
BMI at age 25/(kg/m^2^), (SD)	22.6 (3.2)	22.3 (3.0)	22.0 (2.7)	21.5 (2.5)	21.4 (2.5)
Age at menarche, %					
<15 years old	22.7	19.7	28.9	43.4	58.1
≥15 years old	77.3	80.3	71.1	56.6	41.9
Age at menarche/years old, (SD)	16.0 (2.0)	16.3 (2.1)	15.6 (2.0)	14.9 (1.7)	14.3 (1.6)
Oral contraceptive pills use, %					
Never used	94.7	90.3	87.6	90.2	94.6
Age at first birth, %					
≤21 years old	54.3	45.0	23.9	22.7	24.8
21–25 years old	30.4	38.4	45.5	59.3	52.8
≥25 years old	15.3	16.7	30.6	18.1	22.4
Age at first birth/years old, (SD)	22.0 (3.6)	22.5 (3.4)	24.0 (3.2)	23.5 (2.7)	23.8 (2.9)
Numbers of live births, %					
1	4.4	5.3	36.1	55.9	62.9
2	8.9	27.8	39.2	32.8	31.3
>2	86.7	66.9	24.6	11.3	5.8

The percentages of different breastfeeding behaviors of the first child are presented in Table 2. The mean breastfeeding duration in different age groups were 15.4 (9.2), 14.8 (8.2), 14.7 (8.9), 12.8 (7.2), 13.1 (7.2) months. The percentages of any breastfeeding were over 90% in all age groups. The percentages of breastfeeding at 6 months were over 90% in the 1930s’, 1940s’ and 1950s’ groups, while that in the 1960s’ and 70s’ groups were 89%. The percentages of breastfeeding at 12 months were 81.3, 78.9, 74.1, 65.3 and 65.0% in the 1930s’, 1940s’, 1950s’, 1960s’ and 1970s’ groups respectively. The medians of breastfeeding duration of the first child in all groups were 12 months.

**Table 2:** Different breastfeeding behaviors of the first child in different age groups

	1930–1938	1939–1948	1949–1958	1959–1968	1969–1974	Total
Breastfeeding duration/months, (SD)	15.4 (9.2)	14.8 (8.2)	14.7 (8.9)	12.8 (7.2)	13.1 (7.2)	14.1 (8.2)
Any breastfeeding, %	94.9	95.6	95.6	94.9	95.4	95.3
Breastfeeding at 6 months, %	91.2	92.0	91.8	89.4	89.2	90.9
Breastfeeding at 12 months, %	81.3	78.9	74.1	65.3	65.0	72.2
Median of bf duration/ months	12	12	12	12	12	12

The associations between breastfeeding duration and various socioeconomic factors and reproductive history are presented in Table 3. In urban areas, the age at menarche and BMI at age 25 had positive associations with breastfeeding duration (*p* < 0.0001), while birth year, level of highest education, household size, age at first birth and use of oral contraceptive pills demonstrated negative associations (*p* < 0.0001). In rural areas, the association between breastfeeding duration and various socioeconomic factors and reproductive history was slightly different from that in the urban regions: household size and age at first birth had positive associations (*p* < 0.0001) while others findings remained similar to its urban counterpart.

**Table 3:** Results of multivariate linear regression analysis for the first child breastfeeding duration and various socioeconomic factors

	Urban		Rural	
	β	*p*	β	*p*
Intercept	16.8	< 0.0001	9.9	< 0.0001
Age groups	−0.7	< 0.0001	−0.6	< 0.0001
Highest education^a^				
Middle school	−1.3	< 0.0001	−0.6	< 0.0001
High school& above	−1.6	< 0.0001	−1.2	< 0.0001
Household size	−0.1	< 0.0001	0.2	< 0.0001
BMI at 25 years old	0.4	< 0.0001	0.2	< 0.0001
Age at menarche	0.2	< 0.0001	0.2	< 0.0001
Oral contraceptive pills use	−0.5	< 0.0001	−2.8	< 0.0001
Age at first birth	−0.2	< 0.0001	0.2	< 0.0001

^a^Reference: no formal school & primary school

Model is adjusted for regional difference, age groups, highest level of education, household size, BMI at age 25, age at menarche, use of oral contraceptive pills and age at first birth

**Fig. 1:**
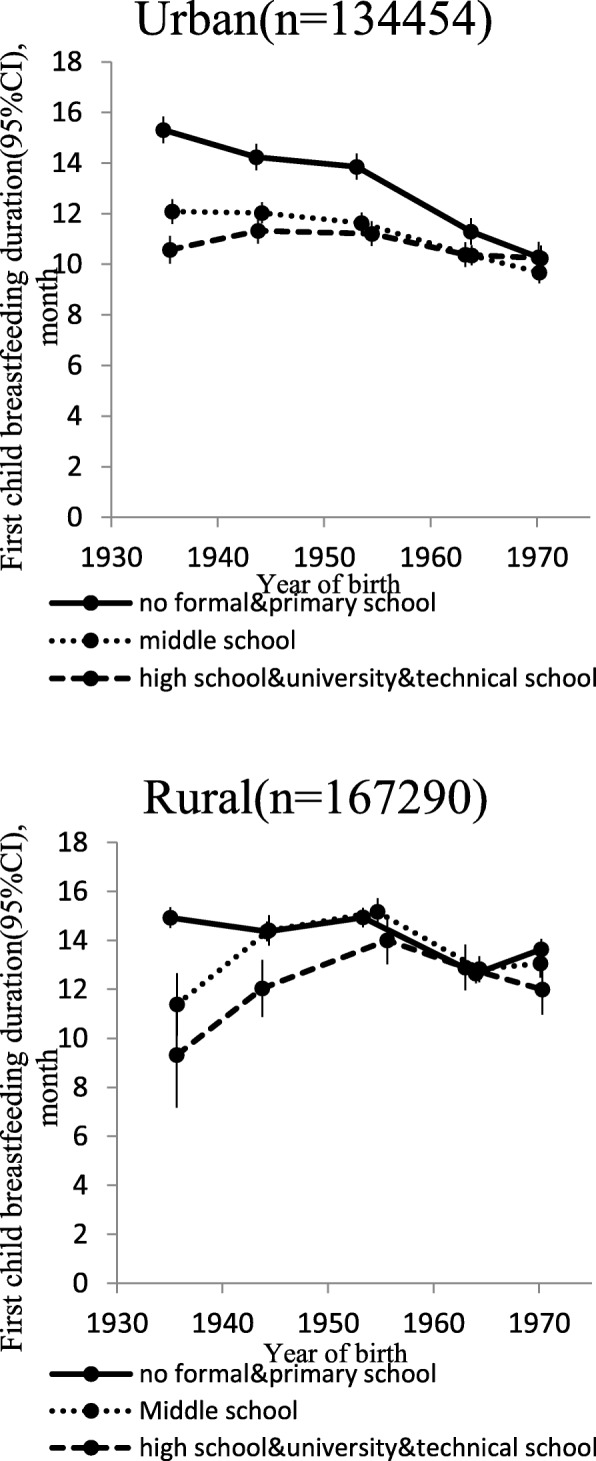
Breastfeeding duration of the first child across different age groups by level of education, among urban and rural areas. Estimates adjusted for age groups, age at menarche, age at first birth, household size, BMI at age 25, use of oral contraceptive pills and regional difference

Figure 1 presents the association between different age groups and breastfeeding duration by level of education. In urban areas, breastfeeding durations of the first child were 15.3, 14.2, 13.8, 11.3 and 10.3 months in women with no formal schooling and with primary school education. Breastfeeding durations were 12.1, 12.0, 11.6, 10.4 and 9.7 months in women with middle school education, and the durations were 10.6, 11.3, 11.2, 10.4 and 10.2 months in women with higher than middle school education. In rural areas, breastfeeding durations were 14.9, 14.4, 14.9, 12.7 and 13.6 months in women with no formal schooling and with primary school education. Breastfeeding durations were 11.4, 14.4, 15.2, 12.8 and 13.1 months in women with middle school education, and the durations were 9.3, 12.0, 14.0, 12.9 and 12.0 months in women with higher than middle school education.

## Discussion

### Main findings

This large cross-sectional study of over 300,000 women from five urban and five rural areas in China provided evidence for the association between different age groups and breastfeeding duration. It also comprehensively assessed the association of breastfeeding duration with different socioeconomic influence in different age groups. To our knowledge, this is one of the first studies in China to examine the associations between breastfeeding duration and different age groups as well as other sociodemographic and fertility related factors. The main findings were that in both urban and rural areas, birth year, level of highest education and use of oral contraceptive pills had negative associations with breastfeeding duration, while BMI at age 25 and age at menarche had positive associations with breastfeeding duration. Household size and age at first birth was negatively associated with breastfeeding duration in the urban areas, while positively associated with breastfeeding duration in the rural regions.

### Explanations

This study shows that younger age groups was negatively associated with breastfeeding duration in both urban and rural areas. Throughout the decades involved in this study, the socioeconomic and cultural environment have greatly improved and changed which may subsequently shape women’s attitudes and behaviors. Such reasons are multifactorial and we refer to the conceptual framework used by Rollins et al. [6] to explore this association within the Chinese context. Breastfeeding determinants are categorized into three domains: structural, environmental, and individual aspects [6].

#### Structural determinants

Structural determinants include both market and sociocultural context, while policy changes may be the driving force that shapes such environment. Policy implementation may significantly contribute to the difference in breastfeeding practice among different age groups. For example, following the Reform and Opening-up Policy, socioeconomic environment rapidly improved, and breastfeeding duration is observed to have declined from the 1950s’ cohort to the 1970s’ cohort [7].

Prevalence of breast milk substitute may be related to this observation, especially in large cities [8]. The presence of infant formula has made it possible for women to reconcile the demands of reproduction and childcare with different roles brought about by increasing gender awareness [9], thus affecting breastfeeding choices and duration.

#### Environmental determinants

Environmental determinants refer to workplace, family and community and the healthcare system and services. Since the institution of the Economic Reform policy in 1978, the labor market has transformed substantially in both urban and rural regions. As state owned enterprises and welfare were decentralized, women during this process struggled between the burden of unemployment or marginalized employment status and domestic demands [10]. At the same time, the large rural and urban migration of millions of people in search for jobs was also a feature of this period. During this reform era, childcare may have yielded to livelihood issues, and may be related to the decline in breastfeeding duration in the 1950s’ and 1960s’ age groups. During the post-reform era, there were great improvement in market economics and the labor market. Under such influence, employment might have become an important factor in breastfeeding-related issues. A research showed that the percentage of female workers reached 50% in 1991 as compared to 36.5% in 1979 in Shenzhen [11], demonstrating the influence of the Economic Reform policy. Yet, the demands of work, short maternity leave and lack of proper supportive environment may prevent women from maintaining breastfeeding after returning to paid work [12]. As a result, women in urban cities became more career oriented, less time was spent at home, and complementary food and breastmilk substitute may be introduced earlier, contributing to the decreasing breastfeeding duration [13–16].

Family and community settings are origins from which childrearing practices are shared. Family structure, thus, offers opportunities for different interactions [17] that may host varying information regarding breastfeeding. After the Family Planning Policy, the rate of couple-only families (i.e.: a family comprised of only the couple) and extended families (i.e.: a family comprised of the grandparents, parents and grandchildren) have gradually increased, while that of the standard nuclear families (i.e.: a family comprised of the couple and their children) have decreased [18, 19]. A survey reported that in the grandmother-mother-grandchildren relationship, although the mothers may decide on the breastfeeding method, the grandmothers tended to intervene using traditional method (i.e.: early use of infant formula and sugar water instead of breastmilk) [20]. Moreover, it is found in China that breastfeeding duration is negatively associated with households living with grandparents [20]. Hence, transformation of family structure may also influence breastfeeding duration.

Medical services also play a pivotal role in maternity care. Studies found that mothers who had had an operative delivery were observed to have lower rates of exclusive breastfeeding [21], while the rate of caesarean section had grown from 1% in the 1950s’ to over 20% in the 1980s’ [22]. The prevalence of operative delivery may have become an important reason for the decline in breastfeeding duration.

The Great Chinese Famine [23] of 1959 through to 1961 may also account for the different breastfeeding duration rates between different age groups. It has been suggested that starvation, poor living environment, lack of healthcare and housing congestion may lead to reduction in breast milk [24]. Hence, the Famine may be associated with the decreased breastfeeding duration observed in the 1939–1948 group [24]. Throughout the decades involved, Chinese mothers of each age group encountered different socioeconomic environment that influence their life experiences and also breastfeeding behaviors.

#### Individual determinants

Education has been identified as a significant factor that has an influence on the acceptance and practice of breastfeeding [25], including a mother’s knowledge, behavior and awareness about nutrition for her newborn [26]. In the present study, it was found that in both urban and rural areas educational attainment was generally improving [27] throughout generations and was inversely associated with breastfeeding duration. This result is supported in a systematic review that described this inverse relationship among Chinese mothers [28]. It is also consistent with previous studies that demonstrates the negative association between education and rate of breastfeeding in the developing countries [23, 29, 30]. As educational attainment improves, women exhibit better adaptability to urbanization, have more opportunities in the workforce and tend to choose career over fertility-related matters [31]. In addition, as educational attainment advances, women began to pursue gender equality and the degree of pursuit was positively associated with educational level [32]. Therefore, women’s attitudes toward gender roles differ throughout the generations [33]. In the context of urbanization brought about by rapid economic development, women with higher educational attainment tend to have better capacity to adapt to such changes. Higher education often results in a better income yet heavier workload frequently encountered in a full time job that prevent her from breastfeeding and resort to infant formula instead [33, 34]. In general, educational attainment is one of the most important individual determinants that influence breastfeeding practices in the five age groups.

The perceived insufficiency of breast milk is another factor affecting mothers’ breastfeeding choices and may be related to the decline in breastfeeding [16], especially in the minority and less developed areas [35].

The difference between urban and rural regions is attributable to the interplay of multiple socioeconomic factors. The degree of implementation of the Family Planning policy was different in the urban and rural regions, and the same historical events may have had different degrees of impact as well [36]. Transitioning of women’s status is delayed, hence opportunities to schooling and employment may not be as accessible as in urban cities. Besides, the economic status in rural and urban regions is different. Women in urban regions mostly hold contract jobs while women in rural areas tend to be in temporary positions. Such difference in type of employment predisposes rural mothers to less supportive resources or welfare [37]. Availability of breast milk substitutes and programs are not as abundant as in the more developed regions. In the context of different times, Chinese mothers lived in different environment and encountered various challenges typical of that generation and could have been associated with breastfeeding behaviors observed in this study [8].

#### Strength and limitations

The baseline of a large cohort study provided a wide range of data on over 300,000 Chinese women from 10 different urban and rural areas. This wide range of data, with very little missing information and good reproducibility, permitted reliable analyses of the trends and relevance to breastfeeding duration and different age groups***.*** This platform enabled this first large-scale study in China that describes the influence of different age groups on breastfeeding duration: it is strongly associated with level of education, location of residency (rural and urban), BMI at age 25, and the age at first pregnancy. The divide of the different age groups also has its own characteristics given the various social, economic and political backgrounds involved.

The present study has several limitations. Firstly, causal inference between different age groups and breastfeeding duration is limited due to the nature of the study being cross-sectional. Prospective studies are needed to provide more insight and to establish causal relation. Secondly, the design of the baseline cohort study was not primarily focused on maternal health, but to collect information on exposures relevant to chronic diseases. We extracted data on reproductive health that may have influenced breastfeeding duration. As a result, there may be missing information on other factors possibly associated with breastfeeding. Thirdly, recall bias is another factor that may cause inadvertent error on breastfeeding duration as mothers were asked to recall breastfeeding duration many years after childbirth. To control for such bias, we used breastfeeding duration of the first child which was more accurate. Fourthly, in later years multi-child families were likely to be biased towards ethnic minorities and rural mothers, where they were able to have more than one child. However, we analyzed the association between birth order and breastfeeding duration and found no significant association between them. Thus, this bias would be acceptable. Finally, we calculated BMI at age 25 by using data of the weight at 25 years old and the present height. Although participants’ heights might have changed through the years, the bias/error induced by this calculation was acceptable since the degree of change was limited.

## Conclusions

This large population-based study demonstrated a negative association between younger age groups and breastfeeding duration in 10 geographically distinct areas in China. It also showed a negative association between breastfeeding duration and level of education. The results have reflected the challenges mothers of different age groups faced. In order to promote breastfeeding in the current and future generations, healthcare providers need to continue advocating, educating and assisting mothers and family members on issues related to breastfeeding. We also call for collaboration with various sectors and concerned groups to actions that promote breastfeeding-friendly environment and measures.

## Abbreviations

BMI: body max index
SD: standard deviation
